# Fitness implications of seasonal climate variation in Columbian ground squirrels

**DOI:** 10.1002/ece3.2279

**Published:** 2016-07-16

**Authors:** F. Stephen Dobson, Jeffrey E. Lane, Matthew Low, Jan O. Murie

**Affiliations:** ^1^ Department of Biological SciencesAuburn UniversityAuburnAlabama36849; ^2^ Department of BiologyUniversity of SaskatchewanSaskatoonSaskatchewanS7N 5E2Canada; ^3^ Department of EcologySwedish University of Agricultural Sciences75007UppsalaSweden; ^4^ Department of Biological SciencesUniversity of AlbertaEdmontonAlbertaT6G 2E9Canada

**Keywords:** Climate, fitness, ground squirrels, seasons, sliding windows

## Abstract

The influence of climate change on the fitness of wild populations is often studied in the context of the spring onset of the reproductive season. This focus is relevant for climate influences on reproductive success, but neglects other fitness‐relevant periods (e.g., autumn preparation for overwintering). We examined variation in climate variables (temperature, rainfall, snowfall, and snowpack) across the full annual cycle of Columbian ground squirrels (*Urocitellus columbianus*) for 21 years. We investigated seasonal climate variables that were associated with fitness variables, climate variables that exhibited directional changes across the study period, and finally observed declines in fitness (−0.03 units/year; total decline = 37%) that were associated with directional changes in climate variables. Annual fitness of adult female ground squirrels was positively associated with spring temperature (*r *=* *0.69) and early summer rainfall (*r *=* *0.56) and negatively associated with spring snow conditions (*r *=* *−0.44 to −0.66). Across the 21 years, spring snowmelt has become significantly delayed (*r *=* *0.48) and summer rainfall became significantly reduced (*r *=* *−0.53). Using a standardized partial regression model, we found that directional changes in the timing of spring snowmelt and early summer rainfall (i.e., progressively drier summers) had moderate influences on annual fitness, with the latter statistically significant (*ρ *= −0.314 and 0.437, respectively). The summer period corresponds to prehibernation fattening of young and adult ground squirrels. Had we focused on a single point in time (viz. the onset of the breeding season), we would have underestimated the influences of climate change on our population. Rather, we obtained a comprehensive understanding of the influences of climate change on individual fitness by investigating the full lifecycle.

## Introduction

The relationship between climate and fitness is a fundamental biological process that influences population dynamics and regulation (Schwartz 2003; Moller et al. 2008). Due to the overwhelming evidence of anthropogenic influences on global climate (IPCC 2014), there has been a renewed research emphasis on these climate‐fitness associations, and further, on how anthropogenic climate change will affect the fitness and population viability of wild animals (e.g., Both et al. 2006, 2009; Moller et al. 2008; Moyes et al. 2011; Tack et al. 2015). This is particularly true in the northern temperate zone, where climate change is expected to be most dramatic (Chambers et al. 2013). Collectively, these studies demonstrate a general pattern of earlier timing of reproduction in response to climate warming that advances the onset of spring (Parmesan 2006; Thackeray et al. 2010; Chambers et al. 2013). However, delays in phenology may also produce detrimental fitness consequences (Lane et al. 2012; Chambers et al. 2013). While intuitive, there is surprisingly little evidence that specifically links climate change to variation in population dynamics and, by extension, viability. Establishing such links requires knowledge of how individual fitness (and the underlying components of reproduction and survival) covaries with climate. As climate change is expected to accelerate in the future (IPCC 2014), such explicit links are arguably more relevant in our establishing an ability to predict responses to climate change than broad‐scale phenotypic shifts (e.g., Jenouvrier et al. 2009; Ozgul et al. 2010).

The primary research focus in studies of ecological responses to climate change has been on the timing of events linked to breeding (e.g., flowering date, spring arrival from migration, emergence from hibernation, or breeding dates; Garant et al. 2008; Møller 2013). This focus is certainly relevant for one component of fitness, reproductive success, but ignores potential influences of climatic variation during other fitness‐relevant periods (e.g., autumnal preparation for overwintering). As climates are expected to change during times of the year other than spring (IPCC 2014), this is potentially important with respect to altered juvenile and/or adult survival during the nonbreeding season, or changes in adult condition prior to the breeding season and associated effects on fecundity. Thus, to fully understand population responses to climate change, we need to consider information from periods outside of the spring breeding/growth periods.

The emergence dates from hibernation of Columbian ground squirrels (*Urocitellus columbianus*) are both sensitive to climate conditions and have fitness consequences (Lane et al. 2012). On the east side of the Rocky Mountains, habitats have not exhibited the usual temperate zone pattern of warmer spring temperatures. Rather, over a 20‐year period, spring temperatures declined by about 2°C and snowmelt was delayed by about 57 days, partly owing to later and more frequent spring snowstorms (Lane et al. 2012). However, conditions in spring are unlikely to be the only climate influence, because this species has a relatively short 3‐month active season, during which both reproduction and fat storage for the subsequent 9 months of hibernation must occur (Young 1990; Dobson et al. 1992). This lifecycle suggests that climate conditions throughout the year might have a substantial impact on the species. For example, rainfall might substantially affect food resource availability (and individual condition) during their short annual activity period, thereby influencing the ability of ground squirrels to accumulate sufficient resources to survive the winter. Conditions of snowfall and insulative snow pack during winter might have an influence on survival, especially for younger individuals, as they will affect the microclimatic conditions of the hibernacula and thus energetic costs during hibernation (Murie and Boag 1984; Dobson and Murie 1987). Such climate influences could act independently or synergistically with the influence of spring snowfall on emergence from hibernation and the timing of breeding for adult females (Lane et al. 2012). Our ability to track the fitness of every female in our population of this sedentary species makes it ideal for investigating the potential effects of climate variables on fitness, during and outside of the usual spring emergence/breeding period.

We used 21 years of intensive monitoring to examine how interannual variation in regularly measured climate variables (rainfall, temperature, snowfall, and snowpack) were related to annual fitness of Columbian ground squirrels. In particular, we analyzed climate variables during all the seasonal periods of the entire annual lifecycle. We used a “sliding window” search procedure (Lane et al. 2012; Mihoub et al. 2012) to identify relevant periods of change during the year in both climate and fitness. We first tested whether and when mean annual fitness within the population was associated with climate variables. Second, we examined whether climate variables that were associated with fitness had changed during the study in a directional manner (e.g., did summers get warmer or cooler during the period?). We examined annual fitness components (survival and reproductive success) in composite as well as separately. Finally, we used the above results to construct a standardized partial regression model to test the relative strength of effects of directional changes of climate during different seasons of the year on ground squirrel fitness.

## Methods

### Study system

We studied Columbian ground squirrels in the Rocky Mountains of Alberta, Canada, in Sheep River Provincial Park (50°38′10″N, 114°39′56″W). Our population occupied a 1.8‐ha subalpine meadow along the Sheep River, at an elevation of ~1550 m a.s.l. The study began in 1992 and continued through 2012 (with 2013 used for calculation of subsequent annual fitness of ground squirrels monitored in 2012). At spring emergence from hibernation, the female population averaged 37 individuals (range = 14–77). All ground squirrels in the study meadow were captured within 3 days after emergence from hibernation, usually in late April and early May, in live traps (13 × 13 × 40 cm, Tomahawk Live Trap Co., Hazelhurst, WI, USA) baited with peanut butter. Individuals were weighed to the nearest 5 g with a spring‐slide scale (Pesola AG, Schindellegi, Switzerland), permanently marked with numbered metal tags in both ears (Monel #1; National Band & Tag Co., Newport, KY, USA) and marked for visual identification with a unique black dye mark (Lady Clairol human hair dye) on the dorsal pelage. Ground squirrels were examined for reproductive condition and released at the location of capture. Visual observations and/or trapping (e.g., with observation of a copulatory plug) were used to confirm the day each female mated, usually 3–5 days after emergence from hibernation.

Most females first mated and reproduced at 2 years of age and continued to breed annually thereafter, up to 13 years old. Mating was followed by approximately 24 days of gestation and 27 days of lactation (Murie and Harris 1982). Mothers kept dependent young in a single nest burrow during lactation, and these burrows were characterized by a single unobtrusive entryway. Nest burrows lacked underground connections to main burrow systems, as evidenced by observations of a single mother entering and leaving them during the lactation period. Litter size at weaning averaged about 3 (range = 1–7; Broussard et al. 2008). As young of the year emerged for the first time above ground from these nest burrows, they were captured, ear tagged, weighed (to the nearest gram), and dye marked as described above. Adult (breeding) females were also caught at this time and examined for evidence of reproductive condition from the presence or absence of mammary tissue and elongated nipples. Visual observations of marked mothers using unique individual nest burrows unambiguously identified the mother and associated litter. After the weaning period, all ground squirrels fattened prior to entering hibernation in early to mid August (Dobson et al. 1992).

Over the course of 21 years (1992–2012), the mean date of emergence of mating females from hibernation was 27 April (mean Julian date = 117.2 ± 6.8 days SD, range = 100–142, *n* = 526 female‐years), and the mean date of mating was 1 May (mean Julian date = 121.0 ± 6.2 days SD, range = 106–143, *n* = 526 female‐years). Of females that weaned litters, the mean date when litters first appeared above ground from natal nest burrows was 21 June (mean Julian date = 171.8 ± 5.9 days SD, range = 157–191, *n* = 390 female‐years).

### Climate data

We obtained the following weather data for Okotoks, Alberta, from the Environment Canada weather archive (http://climate.weather.gc.ca/): daily records of mean air temperature, total rainfall, total snowfall, and depth of snow on the ground (viz. the level of snowpack). Spring snowmelt date was the first snow‐free date in each year, after continuous snow accumulation of at least 5 cm for at least 2 days. Okotoks is 50 km from our study meadow, at an elevation of ~1081 m a.s.l., and is in the same general weather path as our study meadow (viz. nearly due East and reflects the same general changes in climate variables; Lane et al. 2012).

### Analyses

Annual fitness was calculated for breeding adult females as the survival of the individual (1/0 if she did/did not survive to the next spring) plus 0.5 times the number of her young of both sexes that survived to the next spring as subadult yearlings (following Qvarnström et al. 2006). We multiplied each surviving offspring by 0.5 to approximate the mother's genetic contribution to the following generation. Dispersal from the natal range is male biased and occurs when males are just over 1 year old and adult females are suckling the subsequent year's new litter (Wiggett and Boag 1992; Neuhaus 2006). Thus, we estimated survival of juveniles from weaning to their spring emergence from hibernation as yearlings, when there was the least chance of bias from dispersal events. From 1992 to 2012, juveniles had an overall mean annual survival of 43.1% (*n* = 1190 juveniles) from weaning to the next spring emergence, and breeding females of age 2 or more had a mean annual survival of 72.7% (*n* = 535 female‐years) from one spring emergence to the next. To determine the contribution of particular components of annual fitness and associations with climate, we separately examined the number of juveniles that survived from weaning to the following spring, and rates of juvenile and maternal survival to the following spring.

We regressed vectors of estimated mean annual fitness of the ground squirrels onto matrices containing the daily climate data by year, separately for each climate variable. This was performed with a sliding window procedure and time periods that ranged between 10 and 30 days (as in Lane et al. 2012; Mihoub et al. 2012). For example, mean annual fitness was regressed on mean temperature for the first 10‐day period of the year (i.e., 1–10 January). Then, the 10‐day period was advanced a day (i.e., 2–11 January), and the regression was run again, and so on through the last 10‐day period of the year (31 December–9 January of the following year). Next, an 11‐day period was used and the entire procedure was run again, and so on up to 30 days. We plotted the resulting coefficients of determination (*R* ^2^ values) against time periods to visualize the relationship between variables and times, and clear peaks indicated the medians of influential “windows” (i.e., periods) during the year. Daily values included in the best time windows were first checked against average AIC contributions to the regression models that included each particular day (see also Mihoub et al. 2012). For variables where peaks could be seen in these sliding window plots, periods with the highest *R* ^2^ were selected as the most influential on annual fitness, and *r* (the correlation coefficient) was taken as a measure of effect size (Nakagawa and Cuthill 2007) of the relationship between annual fitness and the climatic mean within the selected window. We followed Cohen (1988) in considering small, medium, and large effects at *r* = 0.10, 0.30, and 0.50, respectively. We did not make corrections for multiple tests, because Bonferroni procedures are overly conservative (due to a loss of statistical power; Nakagawa 2004).

The sliding window approach searches for maximum associations between mean annual fitness with weather variables. Some abiotic climate variables may influence the fitness of the ground squirrels yet not show “directional” climate change. Other climate variables could show directional changes among years that would be considered climate change and yet have little influence on the fitness of the ground squirrels. Our goals were to identify the periods of the lifecycle that were most sensitive to climatic variation, determine whether these periods experienced directional patterns over the past 21 years, and therefore reveal any covariance of changes in climate and fitness. We also looked for associations among climate variables that might indicate covariation of more than one climate variable at a time, such as temperature and rainfall. Finally, we used regression analyses (R function lm) and standardized partial regression (sem) to examine the influence of climate changes over time to annual fitness values. A test of variance inflation (vif) was used to indicate whether partial regression coefficients were inflated due to colinearity, a danger when the vif factor exceeds 3.0 (Petratis et al. 1996). These latter and the sliding window statistical procedures were run in R (version 3.0.2; R Core Team 2013).

## Results

### Mean annual changes in fitness and climate

Annual fitness of Columbian ground squirrels averaged 1.32 (±0.45 SD, *n* = 21) during the study, and in a linear regression declined by an average of −0.03 (±0.02 SE, *F*_1,19_ = 3.3, *P* = 0.08) per year from 1992 to 2012. Quadratic regression (after Lane et al. 2012) revealed a linear fitness decline of −0.19 (±0.05 SE, *t* = 3.7, *P* = 0.002) that slowed in final few years of the study (quadratic term of 0.01 ± 0.00 SE, *t* = 3.2, *P* = 0.004; multiple *R* ^2^ = 0.46, *F*_2,18_ = 7.7, *P* = 0.004). Mean annual daily temperature (4.61°C overall) and rainfall (1.03 mm) did not significantly change (temperature increase = 0.76°C over 21 years, *β *= 0.04 ± 0.03 SE, *R* ^2^ = 0.07, *F*_1,19_ = 1.4, *P* =0.26; rainfall decrease = 0.01 mm over 21 years, *β *= −0.00 ± 0.01 SE, *R* ^2^ = 0.00, *F*_1,19_ = 0.0, *P* = 0.96). Changes in mean annual daily snowfall and snow accumulation were slight and did not increase significantly (snowfall increase = 0.16 cm, *β *= 0.01 ± 0.00 SE, *R* ^2^ = 0.15, *F*_1,19_ = 3.3, *P* = 0.09; snow accumulation increase  = 2.93 cm, *β *= 0.14 ± 0.09 SE, *R* ^2^ = 0.12, *F*_1,19_ = 2.5, *P* = 0.13).

### Seasonal climate‐fitness associations

Ground squirrel fitness was strongly associated with variations in a number of climate variables during seasonal periods. Increased temperatures at the time of spring emergence from hibernation (late April to mid May) had strong positive associations with annual fitness, whereas increased temperatures at the beginning of hibernation (late August) had the opposite effect (diminished fitness; Table 1). Precipitation patterns, as well, showed divergent effects on fitness depending on the time of year (and associated lifecycle events). When the ground squirrels were emerging from hibernation, spring snowmelt date was significantly negatively associated with annual fitness. Yet, rainfall had a significantly positive influence on annual fitness just after the time of emergence of young from their natal burrows, when young have just shifted from milk to vegetation forage and mothers begin fattening for hibernation (young emerge in mid June to early July; Dobson et al. 1992). However, rainfall during late summer (late July to mid August), as ground squirrels were going into hibernation, was negatively associated with fitness. Snow accumulation and daily snowfall had consistently negative associations with annual fitness, especially in late winter (mid February to early March) and the spring period of ground squirrel emergence from hibernation before females reproduced (late April to mid‐to‐late May), and for the winter and subsequent spring emergence periods after females reproduced.

**Table 1:** Associations of climate variables and mean annual fitness of Columbian ground squirrels. For each of the years 1992–2012, the climate variable was averaged over the specified dates, except for date of snowmelt. “*r* with annual fitness” indicates the correlation of mean annual fitness on the mean daily value of the climate variable for the 21 years of the study

Variable	Dates	Days	*r* with annual fitness	*r* with year
Temperature	30 April–11 May	12	0.691***	−0.235
Temperature	22 Aug–1 September	11	−0.685***	0.323
Rainfall	28 June–12 July	15	0.559**	−0.525*
Rainfall	28 July–13 August	17	−0.498*	0.034
Snowmelt	Various Julian Dates		−0.471*	0.484*
Snow Accumulation	27 April–24 May	28	−0.517*	0.201
Snowfall before^a^	14 Feb–8 March	23	−0.663**	0.177
Snowfall before^a^	30 April–19 May	20	−0.442*	0.179
Snowfall after^b^	19 Jan–17 February	30	−0.583**	0.248
Snowfall after^b^	26 April–20 May	25	−0.514*	0.212

a Snowfall before annual fitness was measured, reflects influence of snowfall on the timing of reproduction.

b Snowfall after reproduction and in the year for which annual fitness was measured, reflects influence of snowfall on survival of mother and offspring.

*P ≤ 0.05, **P ≤ 0.01, ***P ≤ 0.001.

**Figure 1:**
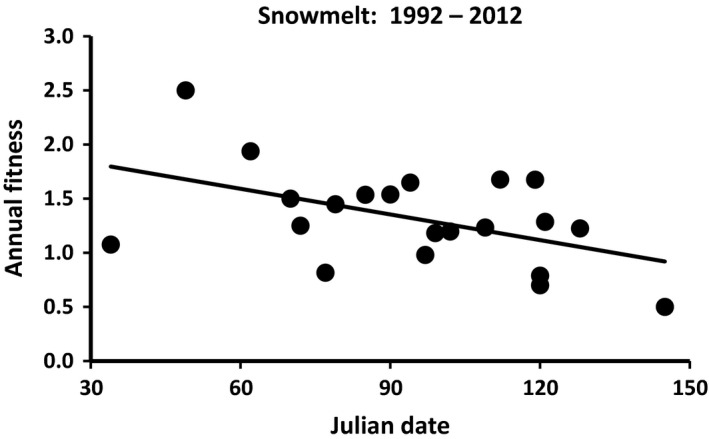
Regression of annual fitness on Julian date of snowmelt. Points represent annual averages between 1992 and 2012.

**Figure 2:**
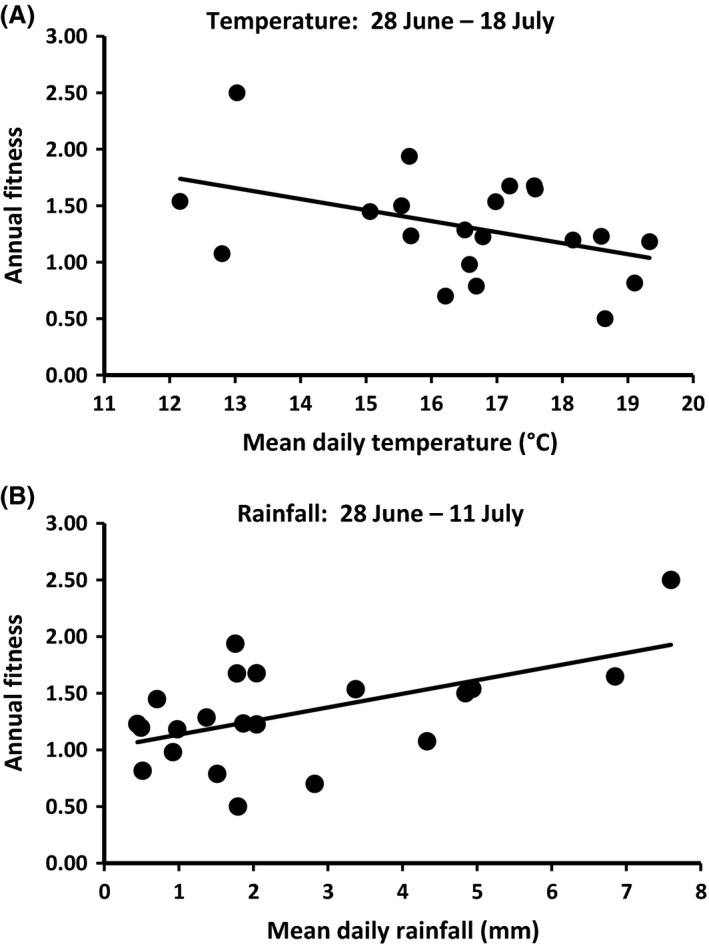
Regression of annual fitness on (A) early summer temperature and (B) early summer rainfall after the young of the year were weaned (late June and July, Table 1), when all ground squirrels were fattening for subsequent emergence into hibernation. Points represent daily period averages for 21 different years.

Except for spring snowmelt date and summer rainfall, none of these combinations of climate variables and periods exhibited significant directional changes over the 21 years of the study (Table 1). The increase in date of snowmelt was associated with a significant decline in annual fitness, of about 0.01 for each day that snowmelt was delayed (Fig. 1; *β *= −0.008 ± 0.003 SE, *R* ^2^ = 0.23, *F*_1,19_ = 5.8, *P* = 0.03). Changes in summer conditions were also associated with significant declines in average annual fitness. Fitness declined by 0.10 for each 1.0°C increase in summer temperature (Fig. 2A; *β *= 0.12 ± 0.04 SE, *R* ^2^ = 0.18, *F*_1,19_ = 4.1, *P* = 0.05) and by 0.12 for each 1.0 mm decrease in average daily summer rainfall (Fig. 2B; *β *= 0.12 ± 0.04 SE, *R* ^2^ = 0.30, *F*_1,19_ = 8.1, *P* = 0.01).

**Figure 3:**
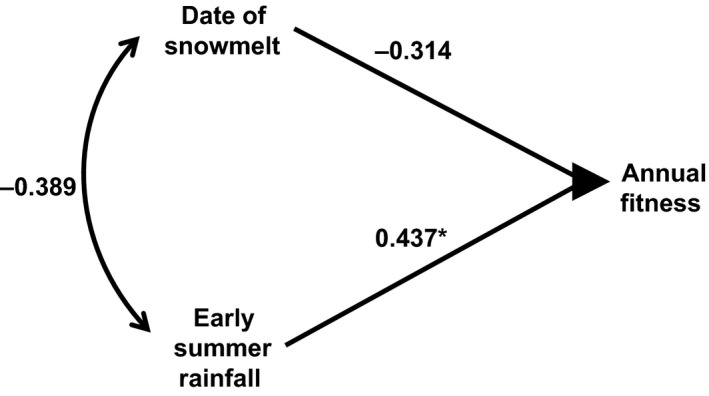
Standardized partial regression analysis of the influence of climate variables that exhibited significant influences on fitness and significant changes over the course of the 21 years of study of a colony of Columbian ground squirrels. A nonsignificant negative correlation between date of snowmelt in late April to late May and mean daily rainfall in late June to mid July is shown (*r* = −0.389, *P* = 0.11, *n* = 21). Relatively, late spring snowmelt and dry early summers were associated with lower annual fitness. Variance inflation due to correlations among independent variables was relatively low (see text). The standardized partial regression coefficient for early summer rainfall was significant (*, *z* = 2.32, *P* = 0.02) and for spring snowmelt was not (*z* = 1.66, *P* = 0.10).

To compare the effects on fitness of the changing seasonal aspects of climate, we examined those climate variables that had both significant influences on annual fitness and changed the most during the past 21 years (Table 1): spring snowmelt date and rainfall in late June to mid July. The standardized partial regression revealed a significant influence of summer climate on annual fitness (Fig. 3), with dry summers leading to lower annual fitness and wet summers leading to greater annual fitness (*P* < 0.05, *n* = 21). Later spring snowmelt dates were associated with diminished fitness, but after statistically accounting for summer rainfall variation, this effect was relatively muted and statistically not significant. There was little variance inflation in the model (vifs  < 1.2).

### Relative impact on different fitness components

Adult female survival and number of surviving juveniles were differentially affected by climate variables. Spring snowmelt date was negatively associated with adult female survival, although not significantly, and significantly negatively associated with number of surviving juveniles and juvenile survival rate (respectively; *r* = −0.380, *P* = 0.09; *r* = −0.477, *P* = 0.01; *r* = −0.447, *P* = 0.04; all *n* = 21). Spring snow accumulation had a stronger negative influence on adult female survival (*r* = −0.566, *P* = 0.007, *n* = 21) than on juvenile survival rate. Mean temperature in late June to mid July (28 June–18 July) was more strongly associated with the number of juvenile ground squirrels that survived their first winter to emerge from hibernation (*r* = −0.431, *P* = 0.05, *n* = 21) than with the survival of mothers to their next emergence from hibernation (*r* = −0.318, *P* = 0.16, *n* = 21). The pattern for rainfall during about the same time period (28 June–11 July) had even stronger associations with the number of juveniles surviving and juvenile survival rate to the following year (*r* = 0.596, *P* = 0.004; *r* = 0.521, *P* = 0.02; both *n* = 21) than with maternal survival to the following year (*r* = 0.297, *P* = 0.19, *n* = 21).

## Discussion

Annual fitness of adult female Columbian ground squirrels declined on average by about 0.03/year over the 21 years of the study (Lane et al. 2012), representing an overall decline of approximately 37%, but mean annual patterns of temperature, rainfall, snowfall, and snow accumulation showed no significant changes. However, as our results show, a focus on mean annual patterns brushes over potentially biologically significant changes on shorter time scales (see also Stopher et al. 2014). Previously, Lane et al. (2012) reported that spring temperatures were declining during the past two decades (although not statistically significantly), indicating that if annual mean temperatures were stable or increasing, then there must be corresponding increases in temperatures at other times of the year. Moreover, across North America the general pattern of temperature increases due to climate change are approximately 2°C over 25‐year periods, particularly in places where sciurid rodents have been studied (Inouye et al. 2000; Réale et al. 2003). Thus, we had reason to suspect strong seasonal changes working in opposite directions to each other. More importantly, Lane et al. (2012) suggested that seasonal climate changes should be associated with changes in mean annual fitness.

Spring snowmelt exhibited significant change over the course of the study, with more frequent late spring snowstorms likely producing subsequent delayed snowmelt. Early summer rainfall also decreased significantly. Both of these seasonal climate patterns were significantly associated with declines in mean annual fitness of adult female ground squirrels. The timing of spring snowmelt was negatively associated with annual fitness, at the time when adult female ground squirrels were emerging from hibernation and mating (Neuhaus et al. 1999; Lane et al. 2011, 2012). Also, early summer rainfall was positively associated with annual fitness, during and just after the weaning of litters, and when mothers and pups began to gain mass before hibernation (late June to mid July). Increased rainfall during these long summer days stimulates growth of grasses and herbs that are essential forage for ground squirrels (Ritchie 1988, 1990; Elliot and Flinders 1991). In particular, forage water content is important for putting on weight in preparation for hibernation (Bintz 1984).

Early summer rainfall influenced mean annual fitness of adult females more strongly than did the time of spring snowmelt. Both of these variables showed significant directional changes over the years of study, with spring snowmelt getting later and early summers getting drier. Thus, while both spring and summer climate conditions might be important to populations of Columbian ground squirrels, drier early summers seemed more important to the general decline in mean annual fitness. While not significant, spring snowmelt and early summer drought exhibited a negative correlation. Thus, in years when snowmelt is late and the early summer is particularly dry, we might expect to see severe declines in population size. This is precisely what occurred in 2002, when the latest spring snowmelt (25 May) was associated with an early summer average rainfall of 1.79 mm/day (compared to the 21‐year mean of 2.52 mm/day), producing a nearly 50% decline in the population (from 51 to 26 breeding females).

There were several climatic influences on fitness that did not show significant seasonal patterns concordant with climate change projections. These included both positive (spring) and negative (late summer) influences of mean daily temperatures, negative effects of late summer rainfall, and negative effects of overwinter and spring snowfalls. These abiotic characteristics could exacerbate fitness declines in future, if current trends in climate change in this part of the Rocky Mountains continue. As well, unusual annual events (i.e., “extreme” climate events; Møller 2013) in any of these variables might produce increments or decrements to fitness and thus population size.

Fitness declines during years of relatively late snowmelt resulted primarily from lower survival of juveniles to the following spring. Spring coincides with mating and the early stages of gestation of adult females, suggesting that early onset of reproduction is important to fitness, likely due to the short growing season for these ground squirrels (Zammuto and Millar 1985; Dobson and Murie 1987). Conditions of rainfall and temperature in early summer appeared to influence both the production and survival of juveniles, with hot and dry summers leading to lower fitness contributions from both lower numbers of offspring and lower survival of these offspring through their first hibernation. Juvenile survival is generally much lower than that of adults and exhibits more pronounced interannual fluctuations (Dobson and Kjelgaard 1985a,b; Dobson and Murie 1987; Dobson 1988, 1995), suggesting that it may be more sensitive to both abiotic and biotic environmental conditions. The timing of reproduction may have had a strong influence on juvenile survival, and thus annual fitness, due to a mismatch between the timing of reproduction and environmental resources (e.g., Both et al. 2009; Cahill et al. 2013; Chambers et al. 2013; Møller 2013). Spring snowfall in the year that followed reproduction was also associated with lowered fitness, resulting in this case primarily from significantly lower survival of breeding females. In general, both adult female survival and juvenile survival contributed to changes in mean annual fitness.

We conclude that climate variables have demonstrable influences on the fitness of Columbian ground squirrels, that some climate variables are changing over time when examined within specific seasonal periods during the year, and that fitness of breeding adult females is influenced by some of these aspects of climate change. The patterns that we found were complicated and went well beyond examination of the spring timing of activity and subsequent reproduction, phenomena that have been the focus of many studies of organismal responses to climate change (reviews by Parmesan 2006; Thackeray et al. 2010; Chambers et al. 2013). Our use of annual fitness included influences on reproduction, both the timing of reproduction in a temperate environment (Lane et al. 2012) and early summer conditions during the period when juveniles become independent of the mother. Our measure of annual fitness included influences on survival, and both maternal and offspring survival were associated with early summer weather conditions. Had we focused only on the breeding period, we would have missed the major impact caused by changes of rainfall and perhaps temperature that occurred later in the active season of the ground squirrels. Future studies of changing climate influences on organisms might benefit from a focus that includes times of the year that fall outside of the period of reproduction.

## Conflict of Interest

None declared.
